# RNA-seq Analysis of Cold and Drought Responsive Transcriptomes of *Zea mays* ssp. *mexicana* L.

**DOI:** 10.3389/fpls.2017.00136

**Published:** 2017-02-07

**Authors:** Xiang Lu, Xuan Zhou, Yu Cao, Meixue Zhou, David McNeil, Shan Liang, Chengwei Yang

**Affiliations:** ^1^Guangdong Provincial Key Laboratory of Biotechnology for Plant Development, School of Life Sciences, South China Normal UniversityGuangzhou, China; ^2^Tasmanian Institute of Agriculture, University of TasmaniaKings Meadows, TAS, Australia; ^3^College of Pratacultural Science, Gansu Agriculture UniversityLanzhou, China; ^4^Dongli Planting and Farming Industrial Co., LTDLianzhou, China

**Keywords:** *Zea mays* ssp. *mexicana* L., cold tolerance, drought tolerance, transcriptome, differentially expressed genes

## Abstract

The annual *Zea mays* ssp. *mexicana* L. is a member of teosinte, a wild relative of the *Zea mays* spp. *mays* L. This subspecies has strong growth and regeneration ability, high tiller numbers, high protein and lysine content as well as resistance to many fungal diseases, and it can be effectively used in maize improvement. In this study, we reported a *Zea mays* ssp. *mexicana* L. transcriptome by merging data from untreated control (CK), cold (4°C) and drought (PEG2000, 20%) treated plant samples. A total of 251,145 transcripts (N50 = 1,269 bp) and 184,280 unigenes (N50 = 923 bp) were predicted, which code for homologs of near 47% of the published maize proteome. Under cold conditions, 2,232 and 817 genes were up-regulated and down-regulated, respectively, while fewer genes were up-regulated (532) and down-regulated (82) under drought stress, indicating that *Zea mays* ssp. *mexicana* L. is more sensitive to the applied cold rather than to the applied drought stresses. Functional enrichment analyses identified many common or specific biological processes and gene sets in response to drought and cold stresses. The ABA dependent pathway, trehalose synthetic pathway and the ICE1-CBF pathway were up-regulated by both stresses. GA associated genes have been shown to differentially regulate the responses to cold in close subspecies in *Zea mays*. These findings and the identified functional genes can provide useful clues for improving abiotic stress tolerance of maize.

## Introduction

In order to survive in nature through evolution, plants developed multiple strategies to adjust to various abiotic stresses by promoting a series of physiological and metabolic processes such as stomatal closure, repression of cell growth and photosynthesis, and activation of respiration (Mishra et al., 2014). Stress induced damages are reduced or removed by improving sugar content, increasing antioxidants, taking mechanical action and inducing molecular chaperone chemicals in plants. The gene regulation networks involved are reviewed elsewhere (Zhu, 2016).

Low temperature and drought stresses are major environmental factors that impact on the geographical distribution and composition of plant species. They can also lead to a decrease in crop quality and productivity (Viswanathan and Zhu, 2002). A number of genes and alternative spliced isoforms responding to these stresses at the transcriptional level have been reported (Leyva et al., 1995; Shinozaki et al., 2003; Thatcher et al., 2016). Transcription factors make up of a large group which extensively involve in these processes (Zhu, 2016). Among them a class of DREB/CBF transcription factors which bind to the DRE/CRT element (A/GCCGAC) play roles in the regulation of the expression of target genes in response to cold and drought in *Arabidopsis* (Nakashima et al., 2009). DREB/CBF-like genes were significantly up-regulated by both stresses of cold and drought (Agarwal et al., 2006). *AaDREB1* from *Adonis amurensis*, as an example, was capable to enhance tolerance to salt, drought, and low temperature in transgenic *Arabidopsis* and rice (Zong et al., 2016). Transcription factors including NAC, bZIP, MYB, and MYC also play important roles in cold and drought stresses. Overexpression of *MlNAC5* (*Miscanthus lutarioriparius*), *SlNAC1* (*Suaeda liaotungensis* K.)*, and VaCBF4 (Vitis amurensis)* enhanced drought and cold stress tolerance of *Arabidopsis*, respectively (Li et al., 2013; Yang et al., 2015; Zong et al., 2016). MAPK related genes that are involved in signal transduction are significantly induced by both cold and drought (Pitzschke et al., 2009). A MAPK gene (GRMZM2G174170_T01) from maize, for example, was activated under cold and drought stresses and was suggested to contribute to stress tolerance in maize (Shan et al., 2013). The expression levels of most *ZmVQ* genes (41 out of 61 members) encoding VQ motif-containing proteins were changed by the drought stress, and half of *ZmVQ* genes were co-expressed with *ZmWRKY* genes in maize (Song et al., 2015).

Phytohormones such as abscisic acid (ABA), ethylene, cytokinin (CK), auxin (IAA), gibberellin (GA), and jasmonate (JA) play important roles in regulating plant growth and development and also in the responses to various biotic and abiotic stresses (Peleg and Blumwald, 2011). ABA synthesis is one of the fastest responses of plants to abiotic stress, triggering ABA-inducible gene expression and causing stomatal closure, thereby reducing water loss via transpiration and eventually restricting cellular growth. 9-cis-epoxycarotenoid dioxygenase (NCED) is a key enzyme in the ABA biosynthetic pathway in several plants which cleaves carotenoids to form the phytohormone ABA (Riahi et al., 2013). Cold or drought stress can induce ABA biosynthesis and exogenous ABA can improve cold or drought tolerance in tomato, *Arabidopsis* and tobacco (Thompson et al., 2000; Wan and Li, 2006; Zhang et al., 2008). In maize, thousands of genes are thought to be involved in abiotic stress. In particular, the gibberellin (GA) metabolic genes. Thus, understanding their expression changes could help to detect molecular mechanisms of the GA pathway under stress conditions (Colebrook et al., 2014). The response of GA metabolic genes to abiotic stress has been investigated and these genes participate in the CBF1-mediated stress-response pathway (Niu et al., 2014) and the GA20ox gene that is responsible for GA biosynthesis is down-regulated by cold treatment in *Zea maize* (Shan et al., 2013).

*Zea mays* ssp. *mexicana* L. is a member of teosintes and a close wild relative of cultivated maize (Almeida et al., 2011). Although teosintes have not yet been widely used in maize breeding, the high genetic diversity shows that *Zea mays* ssp. *mexicana* L. is a genetic reservoir for the improvement of agronomic characteristics of cultivated maize and teosintes (Wang et al., 2008). Since its introduction to China from Japan in 1979, the planting areas of teosinte have increased extensively in southern China, including Guangdong, Fujian, Guangxi, and Sichuan Provinces (Song et al., 2005). As an important forage or silage source, teosinte has a larger plant biomass, higher tiller number and better resistances to various stresses than cultivated maize (Niazi et al., 2015). Similar to maize, most lines of *Zea mays* ssp. *mexicana* L. originate from high altitude in northern and central Mexico and can adapt to acid soil with the best growing temperatures ranging from 25° to 35°C (Fukunaga et al., 2005). It is considered to be a very cold-sensitive crop, especially during the germination and early autotrophic growth stages, despite it having originated at altitudes of about 1,000–2,000 m (Hincha and Zuther, 2014).

RNA-seq (RNA sequencing) has become the most convenient and cost effective tool for understanding gene structure and quantitative transcriptome profiling. It does not depend on the existence of known genomic sequences, and shows a great ability for detecting differentially expressed genes with a broader dynamic range of expression levels (Wang et al., 2009). To discover genetic bases and molecular mechanisms in response to cold or drought in *Zea mays* ssp. *mexicana* L., the RNA-seq technique was employed in this study and the transcriptome of *Zea mays* ssp. *mexicana* L. was presented for the first time. In total, 414,232,462 high quality clean reads were obtained by RNA-seq and were used for *de novo* assembly and annotation of genes from *Zea mays* ssp. *mexicana* L. Differentially expressed genes (DEGs) under cold or drought were also identified. These data will be valuable for the exploration of genetic and molecular mechanisms in response to stresses in *Zea mays* ssp. *mexicana* L. and provide gene resources for breeding programs.

## Materials and methods

### Plant materials and growth conditions

Plump seeds of *Zea mays* ssp. *mexicana* L. variety “8493” were used in this study. Seeds were washed 3 times with distilled water, then soaked in 75% ethanol and 2% sodium hypochlorite for 10 min and 3 min, respectively, and washed 3 times with distilled water before planting in plastic boxes (54 × 28 × 7 cm) containing soil substrates (Jiffy, Netherlands, http://www.jiffygroup.com/jiffy_product_category/substrates/). The boxes were transferred into a climate control box (RXZ 500-C, JIANGNAN Instrument), allowing the seeds to germinate and grow under a photoperiod of 10-h light/14-h dark and a humidity of 60% at 25°C. Thirteen-day-old seedlings were assigned into 3 groups for different treatments. For cold treatment (cold), seedlings were treated for 12 h at 4°C under 10-h light/14-h dark while for drought treatment (drought), seedlings were treated with Hoagland solution containing PEG2000 (20%) for 3 h under the same light condition. Seedlings of the control group (CK) grew under the same photoperiod and were assigned neither of the above treatments. After treatments, fresh tissues (roots, stems and leaves) were sampled and pooled, followed by quick-freezing with liquid nitrogen and storing at −80°C for further analysis. Two replicates for each treatment were applied and plant materials from at least three seedlings were pooled for each sample.

### RNA extraction

Total RNA was extracted using TRIzol® reagent (Invitrogen, Carlsbad, USA) according to the manufacturer's instructions. RNA was treated with RNase-free DNase I (Takara, Japan) to remove any possible DNA. The integrity was checked by gel electrophoresis and the Agilent 2100 bioanalyzer (Agilent Technologies, Palo Alto, CA). Concentration of total RNA was determined using NanoDrop 8000 spectrophotometer (NanoDrop, Wilmington, DE). Total RNA with RIN values ≥7.3 and 28S:18S ratios ≥1.2 were accepted for the following RNA-seq analysis.

### RNA-seq analysis

The Illumina HiSeq2500 platform was applied for RNA-seq analysis. To obtain a comprehensive overview of the *Zea mays* spp. *mexicana* L. transcriptome and the transcript profiles under cold and drought, six libraries were constructed and paired-end sequencing was carried out according to the manufacturer's instructions (Illumina, San Diego, CA). In brief, poly(A)-tailed mRNA was enriched using biotin-Oligo (dT) magnetic beads and fragmented into short fragments of 200–700 bp, followed by converting into double-stranded cDNA. The cDNA fragments were then purified with a QiaQuick PCR extraction kit and adapters were added to both ends of the short fragments. cDNA fragment pools were loaded to Illumina HiSeq2500 platform for sequencing. The generated raw data were processed for further analysis. Library construction and RNA-seq were carried out by Novogene Bioinformatics Technology Co. Ltd, China.

### *De novo* assembly and sequence clustering

The quality of raw data was controlled by using the FastQC tool (http://www.bioinformatics.babraham.ac.uk). Reads containing substantial unknown nucleotides (the proportion of N > 5%) and low-quality reads (>50% of the bases with a quality score ≤ 5) were discarded, and the first 10 bases of processed data were removed. The generated clean data from each sample were merged to perform *de novo* assembly using Trinity with default parameters and an optimized k-mer length of 25. A set of transcripts were generated and served as a reference transcriptome. Based on the shared sequence, transcripts from a gene were clustered into a subclass and the longest transcript was regarded as the unigene. The above processes were performed by Trinity software with default parameters (http://trinityrnaseq.sf.net, updated on 2014/07/17; Haas et al., 2013). To identify possible plant transcription factors (TFs), transcriptional regulators (TRs) and protein kinases (PKs), Plant Transcription factor and Protein Kinase Identifier and Classifier (iTAK) V1.5 was employed to analyse unigenes with the best match result.

Raw Illumina sequences and assembled sequences are available in the Gene Expression Omnibus (GEO) database of the National Center for Biotechnology Information (NCBI) (accession number: GSE76939).

### Identification of differentially expressed unigenes (DEGs)

The paired-reads from each sample were mapped to an assembled reference transcriptome by Bowtie software v1.1.1 with default parameters, and the number of mapped reads were calculated by RSEM (v 2.15) (Langmead et al., 2009; Li and Dewey, 2011). FPKM values were assigned to each unigene, representing normalized expression level by eliminating the influences from library construction and the length of genes (Trapnell et al., 2010). Unigenes with FPKM ≥ 1 were used for further analysis. Fold changes for each unigene under cold or drought condition were determined by comparing the FPKM value to that in control sample, and those unigenes with more than 2-fold change and adjusted *P* < 0.005 were identified as differentially expressed genes (DEGs) by DESeq 2 (V 1.2.10) package of the R statistical program (Love et al., 2014).

### Annotation and function classification of transcripts and unigenes

Annotations were assigned to each unigene based on the top hit in BLASTX search against the protein databases, with the non-redundant (Nr) protein database at GeneBank (http://www.ncbi.nlm.nih.gov) as the highest priority, followed by Swiss-Prot (http://www.expasy.ch/sprot), KEGG (http://www.genome.jp/kegg) and eukaryotic KOG (http://www.ncbi.nlm.nih.gov/KOG) in that priority order. The significant thresholds of *E*-value were set at ≤ 10^−6^. CDS (5′–>3′) predictions of unigenes were confirmed with the priority result of BLAST searched protein.

The accession number and the GI code of the top hit at the BLASTX search against the Nr database were retrieved and ID mapping was performed to obtain GO annotations for the queried unigenes. To further annotate the unigenes, the Blast2GO (v2.5.0) program was employed to get GO annotations based on molecular function (MF), biological process (BP), and cellular component (CC) features (Conesa et al., 2005). GO enrichment analysis were carried out by AgriGO software with FDR < 0.05.

### Quantitative real-time PCR analysis

To further verify the expression profiles of genes in our Illumina RNA-seq data, total RNAs were extracted from three independent groups, which were different from those used for RNA-Seq. First-strand cDNA synthesis and qRT-PCR were carried out with the PrimeScript™ RT reagent Kit with gDNA Eraser (TaKaRa) and TransStart® Tip Green qPCR SuperMix (Transgen), respectively. qRT-PCR was carried out in the BIO-RAD CFX96 sequence detection system according to the manufacturer's instructions. A total of 12 genes were randomly selected and gene-specific primers were designed with online Primer-blast of NCBI. The unigene annotated maize actin-2 was used as the endogenous control (GeneBank accession number NP_001146931.1). Each PCR reaction (20 μL) contained 10 μL 2 × Green qPCR SuperMix, 0.2 μM of each primer and appropriately diluted cDNA. The thermal cycling conditions were 94°C for 30 s, followed by 40 cycles of 5 s at 94°C and 30 s at 60°C then 72°C for 30 s. At the second dissociation stage, 95°C for 10 s, 65°–95°C with increment of 0.5°C for 0.05 s were used. All reactions were performed in triplicate, including the non-template controls. The relative expression level was calculated with the 2^−ΔΔCT^ method (Livak and Schmittgen, 2001).

## Results

### *De novo* assembly and functional annotation of the *Zea mays* ssp. *mexicana* L. transcriptome

To comprehensively understand the transcript profiles of *Zea mays* spp. *mexicana* L. under cold and drought, a total of six samples, including two biological repeats for each condition, were collected and two replicated runs of Illumine sequencing were performed for each sample. Raw reads of 153,219,582, 156,576,828 and 150,941,988 were generated from the control, cold and drought group, respectively. After quality check, the adapters, low-quality sequences and ambiguous reads were removed. A total of 136,233,336 (control), 141,732,126 (cold), and 136,267,000 (drought) clean paired-end reads, corresponding to 17.04 Gb (control), 17.72 Gb (cold), and 17.02 Gb (drought) clean bases, respectively, were used for future assembly. *De novo* assembly was carried out using the Trinity software and 251,145 transcripts and 184,280 unigenes with N50 values of 1,269 and 923 bp were obtained, respectively (Table 1). Approximately 47% of assembled transcripts and 35% of unigenes had a length of more than 500 bp (Supplemental File 1).

**Table 1:** **Overview of the sequencing and assembly of *Zea mays* spp. *mexicana* L**.

**Item**	**Reads**	**Transcripts**	**Unigenes**
Total number of raw reads	460,738,398		
Total number of clean reads	414,232,462		
Total clean nucleotides (bp)	51,779,057,750		
Average Q20 (%)	93.99		
Contig N50 length (bp)		1,269	923
Maximum sequence length (bp)		17,701	17,701
Average length (bp)		776	631
Total number of transcripts/unigenes		251,145	184,280

**Figure 1:**
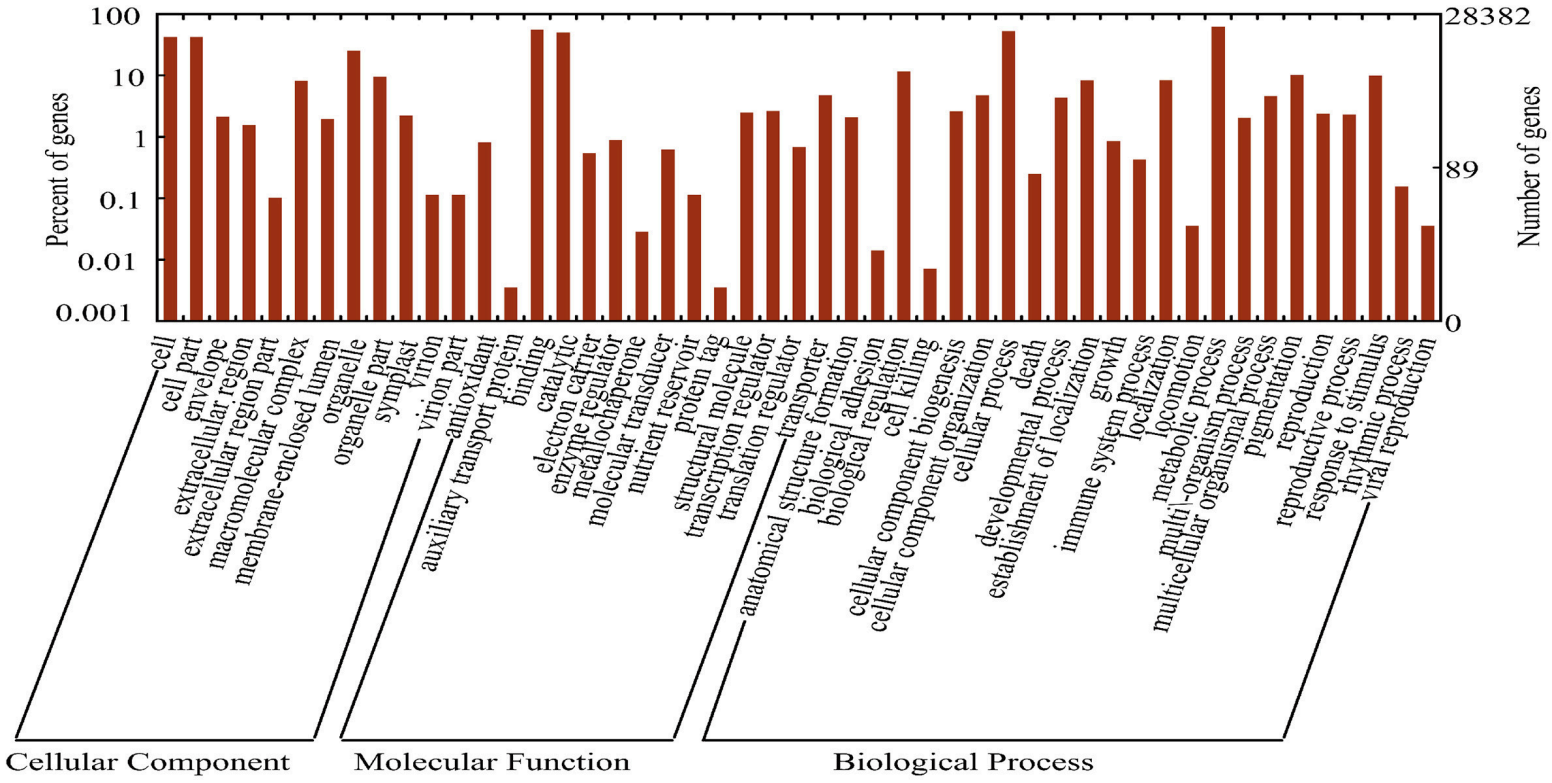
**Functional classification of assembled unigenes**. Functional classification of the assembled unigenes based on Gene Ontology (GO) categorization. GO annotations were assigned to unigenes based on the best BLAST hits, and a total of 98,947 GO terms were retrieved for 28,382 unigenes which were grouped into 49 categories. The left Y-axis represents the percentages of unigenes in each main category. The right Y-axis indicates the numbers of unigenes in each GO category.

Annotations were assigned to each transcript and unigene by BlastX search against the plant protein collections of four public databases (NR, Swiss-Prot, KEGG, and KOG). There were124,297 (49.49%) transcripts and 68,177 (36.7%) unigenes that had at least one significant match (*E* < 1e-6) in one of these databases. Among all annotated transcripts/unigenes, more than 98.5% had at least one hit in the NR database at NCBI, which allowed retrieving GO annotations by the ID mapping method. A total of 28,382 (15.40%) unigenes were assigned to at least one GO term, among which 9769, 5404, and 13,209 unigenes were included into groups of “biological process,” “cellular component,” and “molecular function,” respectively (Figure 1). Among the “biological process” group, 9.96, 7.38, and 7.04% were annotated into “fatty acid biosynthetic process,” “pinoresinol biosynthetic process” and “(1–>3)-beta-D-glucan biosynthetic process,” respectively. In the “molecular function” group the subgroups of “nucleic acid binding” (9.68%), “RNA-directed DNA polymerase activity” (8.4%) and “zinc ion binding” (7.19%) ranked the highest. Additionally, 15.30, 7.49, and 7.48% of unigenes were annotated as cellular components of “integral component of membrane,” “cytoplasm,” and “ribosome,” respectively (Supplemental File 2).

Homologs were also identified by searching against the protein database of the most closed relative *Zea mays* L. and the model species, *Arabidopsis thaliana*. A total of 60,583 and 41,293 unigenes had at least one hit sequence from maize and *Arabidopsis thaliana*, respectively, with an *E* < 1e-6. After removing the replicate records, 27,379 and 15,643 homologs were obtained, representing 46.78 and 44.47% of the maize and *Arabidopsis* proteome, respectively (Supplemental File 3).

A total of 2,157 and 512 unigenes were identified as transcription factors (TF) or transcription regulators (TR) based on the Blast search against the Nr protein database at NCBI, covering 62 TF families and 25 TR families, respectively (Table 2). In addition, predicted protein kinases (PK) that play crucial roles during signal transduction and other biological processes were also obtained, including 1,598 unigenes covering 67 PK families (Table 3).

**Table 2:** **Transcription factor (TF) and transcription regulators (TR) gene families identified from *Zea mays* spp. *mexicana* L**.

**TF Family**	**Number**	**TF Family**	**Number**	**TR Family**	**Number**
C2H2	253	MIKC	12	SNF2	77
bZIP	141	ARF	11	GNAT	52
MYB-related	129	C2C2-YABBY	11	PHD	51
bHLH	125	CPP	11	AUX/IAA	51
zn-clus	123	NF-YB	11	TRAF	47
C3H	112	RWP-RK	11	SET	45
Orphans	109	C2C2-LSD	10	HMG	30
ERF	108	E2F-DP	10	mTERF	26
WRKY	93	PLATZ	10	Jumonji	22
NAC	92	SRS	10	SWI/SNF-BAF60b	18
GRAS	77	Alfin-like	9	IWS1	16
HB	64	BES1	9	LIM	15
C2C2-GATA	53	EIL	9	SWI/SNF-SWI3	13
FAR1	48	AP2	5	Rcd1-like	8
G2-like	44	ARR-B	5	MBF1	8
B3	40	CSD	5	ARID	6
HSF	37	DBP	4	LUG	5
MYB	34	BBR-BPC	3	TAZ	5
C2C2-Dof	33	CAMTA	3	DDT	4
LOB	31	GRF	3	SOH1	3
OFP	29	NF-X1	3	Coactivator p15	3
M-type	28	VOZ	3	MED6	2
SBP	26	C2C2-CO-like	2	MED7	2
TCP	24	LFY	2	RB	2
Trihelix	24	Whirly	2	Pseudo ARR-B	1
TUB	23	DBB	1		
NF-YA	18	HRT	1		
Tify	18	RAV	1		
NF-YC	15	S1Fa-like	1		
zf-HD	14	STAT	1		
GeBP	12	ULT	1		

**Table 3:** **Protein kinase (PK) gene families identified from the *Zea mays* ssp. *mexicana* L**.

**PK Family**	**Number**	**PK Family**	**Number**
SNF1 Related Protein Kinase (SnRK)	132	Leucine Rich Repeat Receptor VIII	11
IRE/NPH/PI dependent/S6 Kinase	104	Leucine Rich Repeat Kinase VII	11
Leucine Rich Repeat Kinase XI & XII	101	Putative protein kinase/Putative receptor-like protein kinase	10
Domain of Unknown Function 26 (DUF26) Kinase	95	APG1 Like Kinase	8
S Domain Kinase (Type 2)	77	Other Kinase	8
Legume Lectin Domain Kinase	76	Receptor Like Cytoplasmic Kinase IV	8
Receptor Like Cytoplasmic Kinase VII	72	Phosphoenolpyruvate Carboxylase Kinase	7
CDC2 Like Kinase Family	71	Putative receptor like protein kinase	7
Unknown Function Kinase	71	Leucine Rich Repeat Kinase X	7
Calcium Dependent Protein Kinase	63	LRK10 Like Kinase (Type 1)	7
MAPK Family	53	Leucine Rich Repeat Kinase IV	7
GmPK6/AtMRK1 Family	36	Receptor Like Cytoplasmic Kinase II	7
Wall Associated Kinase-like Kinase	36	Leucine Rich Repeat Receptor Kinase I & Unknown Receptor Kinase I	6
Casein Kinase I Family	33	Receptor Like Cytoplasmic Kinase I	6
MAP3K	33	Leucine Rich Repeat Kinase I	6
Leucine Rich Repeat Kinase III	31	WNK like kinase - with no lysine kinase	5
MAP2K	29	Possible MAP2K	4
Leucine Rich Repeat Kinase II & X	28	ATN1 Like Family	4
STE20-PAK Like Protein Kinase	26	Ankyrin Repeat Domain Kinase	4
CRPK1 Like Kinase (Types 1 and 2)	25	Leucine Rich Repeat Kinase IX	4
GSK3/Shaggy Like Protein Kinase Family	24	Tousled like kinase	3
Receptor Like Cytoplasmic Kinase VIII	23	RKF3 Like Kinase	3
Receptor Like Cytoplasmic Kinase IX	20	Wall Associated Kinase	3
Plant External Response Like Kinase	20	Leucine Rich Repeat Kinase VI	3
LAMMER Kinase Family	19	Putative protein kinase/Ser_thr kinase like protein/Putative receptor-like protein kinase	3
S Domain Kinase (Type 1)	17	Light Sensor Kinase	2
Receptor Like Cytoplasmic Kinase VI	17	Calcium/Calmodulin Dependent Protein Kinase (CCamK)	2
Receptor Like Cytoplasmic Kinase V	16	Receptor like protein kinase/Receptor lectin kinase like protein	1
Other Protein Kinase	16	Putative LRR receptor-like protein kinase/Receptor protein kinase like protein	1
Crinkly 4 Like Kinase	16	ELM1/PAK1/TOS3 Like Kinase	1
CTR1/EDR1 Kinase	15	C-terminal Ankyrin Repeat Domain Kinase	1
Receptor-like protein kinase	15	Male grem cell-associated kinase (mak)	1
Leucine-rich transmembrane protein kinase/Strubbelig Receptor Family 1	14	C-terminal Ankyrin Repeat Domain Kinase	1
Casein Kinase II Family	12		

### Expression quantification of unigenes and identification of differentially expressed genes (DEGs)

The expression levels of unigenes were evaluated using FPKM values. A total of 43,432 (23.57%), 42,849 (23.25%), 55,928 (30.35%), 48,945 (26.56%), 47,327 (25.68%), and 43,879 (23.81%) unigenes had FPKM ≥ 1 from the sample of control-1, control-2, cold-1, cold-2, drought-1, and drought-2, respectively. However, the percent of unigenes with FPKM ≥ 100 from each sample was less than 1%, ranging from 0.78 to 0.84% (Supplemental File 1). Compared with the control, the cold treatment at 4°C resulted in a larger number of genes with high FPKM values than did the drought treatment with PEG, indicating that the responses induced by cold was more dramatic than that by drought stress in *Zea mays* spp. *mexicana* L. at least with the relative levels of stress used in this trial.

**Figure 2:**
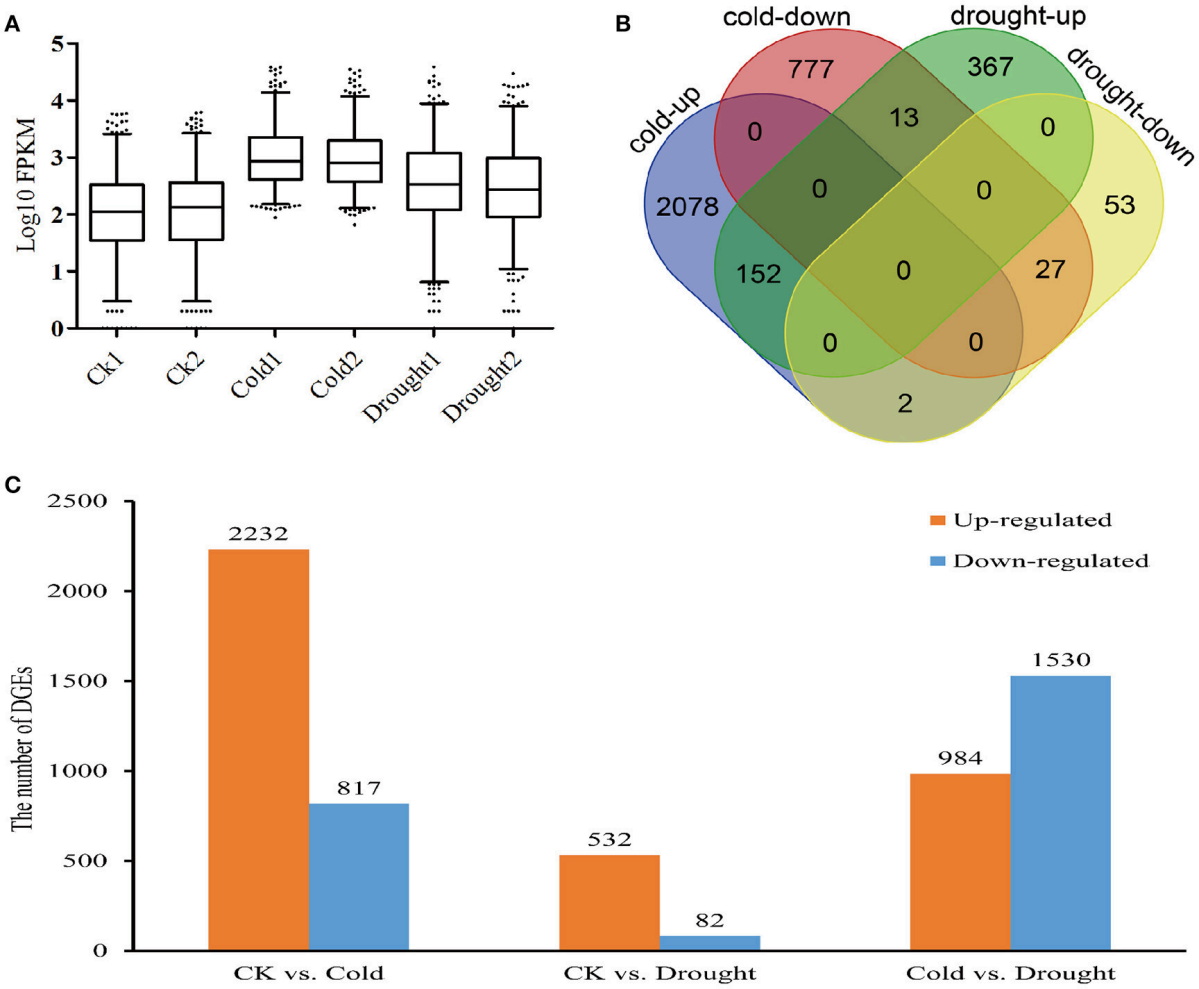
**Gene expressions of differentially expressed genes (DEGs). (A)** Distribution of Log_10_(read number) of DEGs in all tested samples. **(B)** Venn diagram for DEGs in response to cold and drought stress in *Zea mays* ssp. *mexicana* L. **(C)** The numbers of up- and down-regulated unigenes under cold and drought conditions.

To investigate differential responses of *Zea mays* spp. *mexicana* L. to cold or drought stress, data from replicated treatment groups were merged to perform pair-wise comparisons, and unigenes that matched the criteria of |log_2_Change|> = 2 and adjusted *p* < 0.005 were identified as differentially expressed genes (DEGs). Totally, 5,338 DEGs were obtained, with 3,049 and 614 genes differentially responding to cold and drought stresses, respectively. Among those cold-responsive DEGs, 2,232 were up-regulated and 817 were down-regulated (Figure 2C, Supplemental File 4). Notably, annotations for 41.1% of the up-regulated and 25.7% of the down-regulated DEGs were not retrieved from the public maize database (Supplemental File 4), suggesting some special regulation paths in *Zea mays* spp. *mexicana* L. For those DEGs which responded to drought, however, the number of DEGs was less, with only 532 and 82 genes being activated and inhibited by drought, respectively, of which 14.47 (77) and 4.2% (17) were unannotated (Supplemental File 4). The expression level of the whole set of DEGs in all six samples were also evaluated by plotting the Log_10_-transformed FPKM values in descending order (Figure 2A), and the distributions of DEGs' expression suggested activation was present in both cold- and drought-treated samples, with more dramatic transcriptional changes in the cold-treated samples. Taken together, these results indicated that *Zea mays* spp. *mexicana* L. had distinct gene networks in response to cold and drought.

Venn analysis indicated that 194 unigenes were regulated by both factors and 2,855 and 420 were specifically induced by either cold or drought (Figure 2B, Supplemental File 4).Of the 194 shared DEGs, 179 genes had similar response patterns to both cold and drought, including 152 up-regulated genes and 27 down-regulated genes. Two unigenes (c52482_g3 and c52482_g6) similar to protein phosphatase 2C3 and an unannotated unigene (c50928_g2) gave the greatest up-regulation, while the c29390_g1 (thioredoxin H4), c63024_g4 (hypothetical protein ZEAMMB73_373831) and an unannotated unigene (c55190_g2) comprised were the three most down-regulated. Fifteen genes showed the opposite response patterns under cold and drought stresses with 13 genes being down-regulated by cold but up-regulated by drought.

### GO enrichment of DEGs

GO enrichment analysis was performed to investigate over-represented biological processes or molecular functions involved in responses to cold or drought stress. A total of 45 and 89 GO terms were identified based on FDR < 0.05 as over-representations under cold and drought, respectively (Tables 4, 5 and Supplemental File 7).

**Table 4:** **Enriched GO terms corresponding to biological processes and molecular functions under cold stress**.

**GO term ID**	**Term type**	**Term description**	**FDR**
GO:0009408	P	Response to heat	7.40E-10
GO:0009628	P	Response to abiotic stimulus	9.20E-08
GO:0009644	P	Response to high light intensity	9.20E-08
GO:0009266	P	Response to temperature stimulus	3.80E-07
GO:0006355	P	Regulation of transcription, DNA-dependent	3.80E-07
GO:0045449	P	Regulation of transcription	3.80E-07
GO:0051252	P	Regulation of RNA metabolic process	3.90E-07
GO:0031326	P	Regulation of cellular biosynthetic process	9.40E-07
GO:0019219	P	Regulation of nucleobase, nucleoside, nucleotide and nucleic acid metabolic process	9.40E-07
GO:0009889	P	Regulation of biosynthetic process	9.40E-07
GO:0010556	P	Regulation of macromolecule biosynthetic process	9.40E-07
GO:0051171	P	Regulation of nitrogen compound metabolic process	1.20E-06
GO:0009642	P	Response to light intensity	2.30E-06
GO:0030528	F	Transcription regulator activity	2.90E-06
GO:0080090	P	Regulation of primary metabolic process	3.30E-06
GO:0010468	P	Regulation of gene expression	5.60E-06
GO:0031323	P	Regulation of cellular metabolic process	5.60E-06
GO:0006351	P	Transcription, DNA-dependent	6.10E-06
GO:0006350	P	Transcription	6.10E-06
GO:0003700	F	Transcription factor activity	6.50E-06
GO:0032774	P	RNA biosynthetic process	7.40E-06
GO:0060255	P	Regulation of macromolecule metabolic process	1.60E-05
GO:0042542	P	Response to hydrogen peroxide	5.90E-05
GO:0019222	P	Regulation of metabolic process	6.70E-05
GO:0006950	P	Response to stress	7.50E-05
GO:0033926	F	Glycopeptide alpha-N-acetylgalactosaminidase activity	0.00038
GO:0003677	F	DNA binding	0.00046
GO:0000302	P	Response to reactive oxygen species	0.00052
GO:0050794	P	Regulation of cellular process	0.0013
GO:0042221	P	Response to chemical stimulus	0.0019
GO:0016706	F	Oxidoreductase activity, acting on paired donors, with incorporation or reduction of molecular oxygen, 2-oxoglutarate as one donor, and incorporation of one atom each of oxygen into both donors	0.0035
GO:0050789	P	Regulation of biological process	0.0038
GO:0050896	P	Response to stimulus	0.0039
GO:0006560	P	Proline metabolic process	0.005
GO:0065007	P	Biological regulation	0.005
GO:0009416	P	Response to light stimulus	0.0051
GO:0055114	P	Oxidation reduction	0.0053
GO:0009314	P	Response to radiation	0.0085
GO:0006970	P	Response to osmotic stress	0.013
GO:0009415	P	Response to water	0.019
GO:0005509	F	Calcium ion binding	0.023
GO:0016070	P	RNA metabolic process	0.024
GO:0009753	P	Response to jasmonic acid stimulus	0.027
GO:0009414	P	Response to water deprivation	0.032
GO:0042401	P	Cellular biogenic amine biosynthetic process	0.045

P-biological process; F-molecular function

**Table 5:** **Enriched GO terms corresponding to biological processes and molecular functions under drought stress**.

**GO term ID**	**Term type**	**Term description**	**FDR**
GO:0009415	P	Response to water	4.4E-12
GO:0004722	F	Protein serine/threonine phosphatase activity	3.9E-11
GO:0009408	P	Response to heat	4.1E-11
GO:0009414	P	Response to water deprivation	8.6E-11
GO:0006470	P	Protein amino acid dephosphorylation	2E-10
GO:0009628	P	Response to abiotic stimulus	7.3E-10
GO:0009644	P	Response to high light intensity	7.1E-09
GO:0004721	F	Phosphoprotein phosphatase activity	9.3E-09
GO:0003700	F	Transcription factor activity	9.3E-09
GO:0009266	P	Response to temperature stimulus	2.6E-08
GO:0042221	P	Response to chemical stimulus	2.9E-08
GO:0042578	F	Phosphoric ester hydrolase activity	5.6E-08
GO:0030528	F	Transcription regulator activity	5.6E-08
GO:0016791	F	Phosphatase activity	6E-08
GO:0009738	P	Abscisic acid mediated signaling pathway	6.3E-08
GO:0071215	P	Cellular response to abscisic acid stimulus	4.6E-07
GO:0006950	P	Response to stress	1.7E-06
GO:0009642	P	Response to light intensity	2.7E-06
GO:0016311	P	Dephosphorylation	2.7E-06
GO:0009737	P	Response to abscisic acid stimulus	6.5E-06
GO:0042542	P	Response to hydrogen peroxide	0.000021
GO:0050896	P	Response to stimulus	0.000034
GO:0006350	P	Transcription	0.000037
GO:0006351	P	Transcription, DNA-dependent	0.000037
GO:0032774	P	RNA biosynthetic process	0.00004
GO:0016161	F	Beta-amylase activity	0.000052
GO:0006355	P	Regulation of transcription, DNA-dependent	0.000064
GO:0045449	P	Regulation of transcription	0.000064
GO:0051252	P	Regulation of RNA metabolic process	0.000067
GO:0031323	P	Regulation of cellular metabolic process	0.000084
GO:0010556	P	Regulation of macromolecule biosynthetic process	0.000094
GO:0050794	P	Regulation of cellular process	0.000094
GO:0019219	P	Regulation of nucleobase, nucleoside, nucleotide and nucleic acid metabolic process	0.000099
GO:0016160	F	Amylase activity	0.00011
GO:0051171	P	Regulation of nitrogen compound metabolic process	0.00012
GO:0031326	P	Regulation of cellular biosynthetic process	0.00014
GO:0009889	P	Regulation of biosynthetic process	0.00014
GO:0009788	P	Negative regulation of abscisic acid mediated signaling pathway	0.0002
GO:0000302	P	Response to reactive oxygen species	0.00021
GO:0080090	P	Regulation of primary metabolic process	0.00025
GO:0009968	P	Negative regulation of signal transduction	0.00025
GO:0023057	P	Negative regulation of signaling process	0.00025
GO:0010648	P	Negative regulation of cell communication	0.00025
GO:0010468	P	Regulation of gene expression	0.00025
GO:0009416	P	Response to light stimulus	0.00026
GO:0048585	P	Negative regulation of response to stimulus	0.00029
GO:0019222	P	Regulation of metabolic process	0.00029
GO:0016052	P	Carbohydrate catabolic process	0.00035
GO:0009314	P	Response to radiation	0.00036
GO:0006979	P	Response to oxidative stress	0.00037
GO:0060255	P	Regulation of macromolecule metabolic process	0.00038
GO:0009311	P	Oligosaccharide metabolic process	0.00042
GO:0050789	P	Regulation of biological process	0.00045
GO:0043687	P	Post-translational protein modification	0.001
GO:0010029	P	Regulation of seed germination	0.0011
GO:0009787	P	Regulation of abscisic acid mediated signaling pathway	0.0011
GO:0065007	P	Biological regulation	0.0012
GO:0009719	P	Response to endogenous stimulus	0.0012
GO:0006796	P	Phosphate metabolic process	0.0012
GO:0006793	P	Phosphorus metabolic process	0.0012
GO:0009725	P	Response to hormone stimulus	0.0015
GO:0003677	F	DNA binding	0.0017
GO:0000272	P	Polysaccharide catabolic process	0.002
GO:0032870	P	Cellular response to hormone stimulus	0.0024
GO:0005509	F	Calcium ion binding	0.0032
GO:0009755	P	Hormone-mediated signaling pathway	0.0033
GO:0048522	P	Positive regulation of cellular process	0.0038
GO:0006464	P	Protein modification process	0.0039
GO:0016788	F	Hydrolase activity, acting on ester bonds	0.0041
GO:0071495	P	Cellular response to endogenous stimulus	0.0042
GO:0010033	P	Response to organic substance	0.0076
GO:0050793	P	Regulation of developmental process	0.0096
GO:0016070	P	RNA metabolic process	0.0098
GO:0043412	P	Macromolecule modification	0.013
GO:0051239	P	Regulation of multicellular organismal process	0.014
GO:0009845	P	Seed germination	0.02
GO:0005975	P	Carbohydrate metabolic process	0.021
GO:0004553	F	Hydrolase activity, hydrolyzing O-glycosyl compounds	0.021
GO:0016567	P	Protein ubiquitination	0.022
GO:0000151	C	Ubiquitin ligase complex	0.025
GO:0006970	P	Response to osmotic stress	0.026
GO:0071310	P	Cellular response to organic substance	0.027
GO:0032446	P	Protein modification by small protein conjugation	0.028
GO:0048518	P	Positive regulation of biological process	0.028
GO:0004842	F	Ubiquitin-protein ligase activity	0.032
GO:0006629	P	Lipid metabolic process	0.033
GO:0019787	F	Small conjugating protein ligase activity	0.037
GO:0070887	P	Cellular response to chemical stimulus	0.038
GO:0016798	F	Hydrolase activity, acting on glycosyl bonds	0.043

P-biological process; F-molecular function

Apparently, terms related to the response to abiotic stimuli, especially those to temperature and light, were significantly enriched in cold-treated *Zea mays* spp. *mexicana* L. seedlings. Genes for heat shock factor proteins (HSF) such as hsf1 (c59237_g1), hsp3 (c60986_g3), hsf4 (c46749_g1), hsf7 (c54684_g2), hsf26 (c46143_g1), hsp81 (c56395_g1), and hsp101 (c58173_g1) were down-regulated under cold stress. With respect to light responsive genes those for phosphatase 2C50 (c57868_g1) and chloroplastic-like stress response protein (c62415_g1) were up-regulated under cold stress. Additionally, terms related to the regulation of gene expression, mostly at the level of transcription, were over-represented. Several genes for encoding transcription factors, AP2/EREBP (c48755_g1 and c45827_g1), NAC (c60421_g1 and c47289_g2), bZIP (c61581_g3 and c59299_g1), WRKY (c59902_g1, c38115_g1 and c63357_g2 as examples) and ethylene-responsive transcription factor (c56755_g1 and c63899_g2), were up-regulated under cold stress. Together, these observations emphasized the roles of transcriptional regulation of cold-related responses in this species.

For the drought associated responses, however, there were a few differences. Under drought stress, DEGs were enriched for a total of 89 processes. Responses to stimuli, especially those to water deprivation, and regulation at the level of transcription were the most dramatic. Genes for the response to water deprivation were up-regulated under drought. These genes include dehydration-responsive element-binding protein 1D (c49699_g2 and c55715_g7), galactinol synthase gene (c60364_g1) and aquaporin PIP1-5 (c56750_g3 and c35748_g1). Genes involved in abscisic acid responses, such as EID1-like F-box protein (c57232_g1, c57232_g2 and c57232_g3), NAC domain transcription factor (c52045_g1), protein phosphatase 2C (c57868_g1, c50507_g1 and c60116_g4), and stachyose synthase (c55229_g2), were up-regulated under drought.

To further identify the differences between responses induced by cold and drought in *Zea mays* spp. *mexicana L*., a direct comparison was carried out between cold and drought-treated samples. A total of 2,514 assembled unigenes were identified by |log_2_change|> = 2 and adjusted *p* < 0.005 (Figure 2C). GO enrichment analysis for these DEGs indicated that the differences between cold and drought induced responses were in relation to abiotic stresses of temperature and water, transcriptional regulation and protein phosphorylation processes (Supplemental File 5). The FDR value in relation to the abscisic acid related signaling pathway was 0.011 which was very close to the criteria of 0.01 (Table 5), suggesting that this pathway was also a major difference between the two datasets. Together, the enrichment patterns identified by directly comparing responses to cold and drought were in agreement with the above results obtained from comparing each in turn with the untreated control.

### qRT-PCR verification

**Figure 3:**
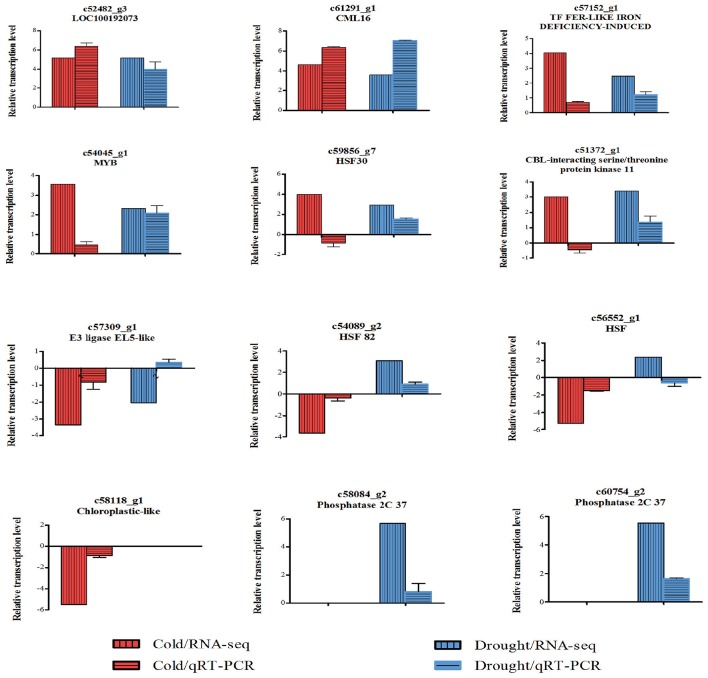
**Verification for expressions of 12 selected DEGs**. Relative expressions of the 12 genes in cold- or drought-treated *Zea mays* spp. *mexicana* L. seedlings were tested by qRT-PCR. Transcription levels were normalized to that of actin and compared to the untreated control. Values of fold change are showed in mean ± SEM. Three independent replicates were performed for each gene. Fold changes of these genes detected in RNA-Seq are also showed for reference. Relative transcription levels were calculated by log2^−ΔΔCT^ method. The names of the closest putative orthologs are indicated for each gene, and the annotations are as following: c52482_g3, uncharacterized protein LOC_100192073; c61291_g1, calcium-binding protein CML16; c57152_g1, transcription factor FER-LIKE IRON DEFICIENCY-INDUCED; c54045_g1, MYB DNA-binding domain superfamily protein isoform X1; c59856_g7, heat shock factor protein HSF30; c51372_g1, CBL-interacting serine/threonine-protein kinase 11 isoform X1; c57309_g1, E3 ubiquitin-protein ligase EL5-like; c54089_g2, heat shock protein 82; c56552_g1, 17.0 kDa class II heat shock protein; c58118_g1, photosystem I reaction center subunit N, chloroplastic-like; c58084_g2, protein phosphatase 2C37; c60754_g2, homeobox-leucine zipper protein ATHB-6.

In order to validate the RNA-seq results, 12 DEGs with different expression patterns were selected for qRT-PCR analysis using gene-specific primers (Supplemental File 6). Among these DEGs, nine expressed under both cold and drought stresses, one regulated by cold only and two regulated by drought only were included. Eight of 10 cold-responsive DEGs and night of 11 drought-responsive DEGs were confirmed by qPCR (Figure 3).

## Discussion

RNA-Seq technology has been successfully used at the level of transcriptomes or whole genomes analysis in many plants and crops (Lu et al., 2010; Xia et al., 2011; Xu et al., 2012, 2014; Zhao et al., 2012; Garcia-Seco et al., 2015). *Zea mays* ssp. *mexicana* L. is a subspecies of *Zea mays*, and it has a genome size of 2.58 Gb (2n = 20) which is more than 20 times greater than the *Arabidopsis thaliana* (0.115 Gb) genome, 6 times greater than the rice (0.43 Gb) genome and equivalent to the *Zea mays* ssp. *mays* genome (2.73 Gb) (Hufford et al., 2012). The variety 8493, which serves as a representative of *Zea mays* spp. *mexicana* L., is reported to grow well under 25°C and can adapt to semi-arid and semi-humid environments (Huang et al., 1992). In this study, RNA-seq analyses were performed for *de novo* assembly of the transcriptome of *Zea mays* spp. *mexicana* L. and identification of the differentially expressed genes (DEGs) in response to cold or drought stress.

### An overview of the *Zea mays* ssp. *mexicana* L. transcriptome

By pooling the clean reads from all samples, we obtained a limited transcriptome which provided a profile the transcript dynamics under cold and drought stresses. The transcriptome identified 184,280 unigenes, and 251,145 transcripts which Blast analysis divided in protein coding and other genes. When growing under cold (4°C) condition, the change of gene expression was dramatic at the global level of transcriptome (Figures 2A,C), indicating that cold induces an extensive activation of transcription. Moreover, GO analysis (Table 4) suggested there was an activation of gene networks involving in cold/heat-induced responses. The drought stress used here, however, induced fewer transcriptional changes, (only 15% as many) than cold (Figure 2C). These observations conformed the geographic distribution and the ideal growth conditions of *Zea mays* ssp. *mexicana* L. (Huang et al., 1992; Hufford et al., 2012). Similar observations have also been found in the elite maize inbred line Zheng 58 of *Zea mays* in which it was reported that the number of differentially expressed genes under drought stress was approximate 42% of that under cold (Shan et al., 2013), suggesting that the more sensitive response to cold rather than drought would be a conserved mechanism in *Zea*.

It is well known that gene expression in response to cold stress is somewhat different from that to drought stress (Shinozaki et al., 2003). In this study, only 194 DEGs are shared in stressed *Zea mays* ssp. *mexicana* L., (6.4 and 31.1% of cold- and drought-related DEGs, respectively). Among these, nearly 90% are regulated in a similar manner by both stresses, while only 15 DEGs are oppositely regulated (Supplemental File 4), indicating that there is a shared network to regulate the cold and drought induced responses in *Zea mays* ssp. *mexicana* L. On the other hand, specific regulations in response to cold or drought stress are also clearly visible in *Zea mays* ssp. *mexicana* L. The DEGs in response to cold are mostly enriched in processes of “Response to abiotic stimulus (GO:0009628),” especially the “Response to heat (GO:0009408),” and “Response to high light intensity (GO:0009644)” (Table 4), while the processes of “Response to water (GO:0009415),” “Response to water deprivation (GO:0009414),” and “protein amino acid dephosphorylation (GO:0004722)” are dominant in the collection of drought-induced DEGs (Table 5).

### Transcriptional regulatory networks and signaling pathways involved in cold and drought induced responses in *Zea mays* ssp. *mexicana* L.

Transcription factors play crucial roles in the regulation of target gene expression by specifically binding to cis-acting elements in the promoter regions (Agarwal et al., 2006; Van Buskirk and Thomashow, 2006). In this study, WRKY, CBL, MYB, NAC, bHLH, bZIP, and AP2/EREBP families that are known to be involved in stress tolerance in plants were identified. Members of WRKY (c56800_g2 and c56800_g1), CBL (c51372_g1 and c49176_g1), MYB (c54045_g1), bHLH (c48928_g1), bZIP (c59299_g1), AP2/EREBP (c51992_g7 and c48755_g1) family are up-regulated by both stresses of cold and drought (Table 2), suggesting shared upstream pathways for signal transduction and regulation under these stimuli. From those down-regulated TF proteins, only one NAC (c57823_g1) protein is consistently down-regulated by both stresses, which is similar to the findings in *Arabidopsis* and rice (Lu et al., 2007; Takasaki et al., 2010) and suggests a negative regulation mechanism in *Zea mays* ssp. *mexicana* L. It is notable that some previously uncharacterized TFs are significantly up- or down-regulated in response to cold or drought only. For example, a member of TUB family (c61146_g4), was found in cold-induced DEGs set but not in the drought-induced set. This is similar to what has been observed in *V. amurensis* (Xu et al., 2014). In addition, some members in the ULT (c53036_g1), BIM2 (c64573_g2), and DBP (c51832_g3) families are found to be up-regulated by cold only (Table 2). The AP2/ERF transcription factor family comprises four major subfamilies, namely AP2, ERF, RAV, and dehydration-responsive element binding proteins (DREB/CBF) (Shinozaki et al., 2003). The roles of DREBs/CBFs in stresses induced response have been well established in numerous plants including *A. thaliana* (Ma et al., 2014), rice (Challam et al., 2015), maize (Liu et al., 2013), and soybean (Kidokoro et al., 2015). The DREB1s/CBFs can activate the downstream cold-responsive genes via specific binding to the DRE/CRT cis-acting element in their promoters, and expressions of these genes are positively correlated with cold tolerance (Mizoi et al., 2012). In *Zea mays* ssp. *mexicana* L., three DREB genes (c51992_g7, c49699_g1 and c49699_g2) were found to be significantly induced by both cold and drought stresses, suggesting that these proteins might functionally act in *Zea mays* ssp. *mexicana* L. during cold and drought stresses.

Signaling components such as kinases participate in numerous processes, including cell division, developmental programs, hormonal responses, and drought, salinity and reactive oxygen species (ROS) signaling responses (Wang et al., 2015). In particular, the MAPK cascade, serving as a signaling joint for various abiotic stress, positively regulates cold acclimation in plants (Chinnusamy et al., 2010). In *Arabidopsis*, the AtMEKK1/ANP1 (MAPKKK)-AtMKK2 (MAPKK)-AtMPK4/6 (MAPK) mitogen-activated protein kinase cascade is necessary for cold acclimation, and AtMKK2 (MAPKK) was identified as a key signal transducer. In addition, overexpression of AtMKK2 induces the cold responsive genes CBF2 and CBF3 and improves cold tolerance in *Arabidopsis* (Teige et al., 2004). In this study, one unigene coding for a MAP kinase (c14147_g1) and another one for a MAPKK protein (c62016_g1) were up-regulated by cold stress only, suggesting that the MAPK cascade may be involved in response to cold in *Zea mays* ssp. *mexicana* L.

### ICE1-CBF pathway genes involved in cold and drought stresses

Cold induced responses come down to different metabolic pathways, gene regulations and cell compartments. The ICE1-CBF-COR transcriptional cascade is a highly conserved module which is activated in response to cold stress (Zhu et al., 2007; Lissarre et al., 2010), and has been demonstrated to have essential roles in response to cold, drought, and other abiotic stresses (Zhou et al., 2011; Peng et al., 2015). ICE1 is a MYC-type transcription factor that binds to the MYC recognition cis-elements (CANNTG) in the promoter of CBF3/DREB1A protein to activate the expression of CBF3/DREB1A, which consequently regulates 40% of COR genes and 46% of cold-regulated transcription factor genes (Lee et al., 2005). In *Arabidopsis*, three CBF proteins, namely CBF1, 2, and 3, are induced by low temperature but not by abscisic acid (ABA) or dehydration (Medina et al., 1999). *CBF1* and *CBF3* can respond to cold at the same time, but the response of the *CBF2* lags behind. *CBF2* negatively regulates the expression of *CBF1* and *CBF3* during cold acclimation, while *CBF1* and *CBF3* are not involved in regulation of other *CBF* genes. CBF1/CBF3 have lower cold acclimation ability and play a subsidiary function in the CBF/DREB1 pathway and cold acclimation (Gilmour et al., 2004; Novillo et al., 2007; Kim et al., 2015). Differences in the functions of CBFs in mangrove *Avicennia marina*, European bilberry (*Vaccinium myrtillus*) and *Brassica rapa* have been observed. For example, *CBF4* is regulated by drought and ABA but not by cold (Haake et al., 2002; Lee et al., 2012; Oakenfull et al., 2013; Peng et al., 2013). In this study, two putative ICE1 homologs (c60792_g1, c13919_g1) were identified in this subspecies. However, expressions of these genes were not affected by cold. A possible explanation is that the ICE1 homolog may be induced rapidly and transiently, and is attenuated in a short time, making it undetectable. In addition, CBF6 has been found to play a role in freezing tolerance in *Festuca pratensis* (Ergon et al., 2016). In our study, a CBF6 (c57018_g4) homologous gene was found to be up-regulated in response to cold and drought stresses, suggesting that this CBF6 homologous gene might play roles in stress resistance in *Zea mays* ssp. *mexicana* L.

### Trehalose may be a common regulator of the response to cold and drought stresses in *Zea mays* ssp. *mexicana* L.

Trehalose is widely found in various organisms and is a non-reducing disaccharide formed by linking two glucose units with an a,a-1,1-glycosidic linkage (Wang et al., 2005). The synthetization of trehalose involves two-steps. The first step is the formation of trehalose-6-phosphate (T6P) from UDP-glucose and glucose-6-phosphate, which is catalyzed by trehalose-6-phosphate synthase (TPS). The second step is the generation of trehalose through dephosphorylation which is catalyzed by trehalose-6-phosphate phosphatase (TPP) (Zang et al., 2011). The genes coding for the TPS and TPP enzymes are thus important for the regulation of trehalose production (Ilhan et al., 2015). Transgenic plants harboring TPS or TPP genes have enhanced tolerance to abiotic stresses by stabilizing dehydrated enzymes, as well as by protecting protein and cellular membranes structures (Nuccio et al., 2015). In our study, a key trehalose-6-phosphate phosphatase (TPP) gene (c45624_g2) was also found to be up-regulated under both cold and drought stresses in *Zea mays* ssp. *mexicana* L. This is in agreement with the positive roles of trehalose in tolerating low temperature and drought stresses.

### Phytohormone dependent pathways in *Zea mays* ssp. *mexicana* L.

Plant hormones play vital roles in adaption to stressful environments by regulating growth, development, nutrient allocation and source/sink transitions. Previous studies showed that different types of plant hormones, including abscisic acid (ABA), cytokinins (CKs), jasmonic acid (JA), gibberellins (GAs), brassinosteroids (BRs), ethylene (ETH), and auxin, have functions in regulating freezing tolerance by CBF-dependent or -independent pathways (Shi et al., 2015). ABA is the key phytohormone used in plants for regulation of the responses to abiotic stresses. As an example, the secondary hyperosmotic stress derived from drought or salt stress causes the accumulation of ABA which in turn rapidly elicits downstream responses in plants (Zhu, 2016). ABA accumulation can cause stomatal closure, thereby reducing water loss and eventually restrict cellular growth. At the molecular level, ABA regulates gene transcription, protein synthesis, signaling pathways, ion transport (and the transport of other organic molecules) and the production of important protectants against dehydration and photoinhibition (Riahi et al., 2013). Cold stress is also reported to induce ABA biosynthesis, and the exogenous application of ABA improves the cold tolerance of plants (De Zelicourt et al., 2016). ABA biosynthesis is one of the important steps used by plants in responding to stresses via the ABA-dependent pathway. The 9-cis epoxycarotenoid dioxygenase 1 (NCED1) is regarded as a key rate-limiting enzyme in ABA biosynthesis. It was originally identified from maize *viviparous 14* mutants, and is responsible for cleavage of the ABA precursor C40-cis-epoxycarotenoids, to either 9-cis-neoxanthin, 9-cis-violaxanthin or both to produce xanthoxin, the direct C15 precursor of ABA (Raghavendra et al., 2010; Boursiac et al., 2013). In *Zea mays* ssp. *mexicana* L., a homolog of NCED1 (c63716_g5) gene was identified. It was up-regulated by drought (Supplemental File 4), which is consistent with results reported previously in *Arabidopsis* (Behnam et al., 2013) and *Phaseolus vulgaris* L. (Qin and Jan, 1999), suggesting conserved regulation of this drought induced response in *Zea mays* ssp. *mexicana* L. It is notable that this NCED1 gene was also activated by cold stress (Supplemental File 4), which is similar to recent observations for OsNCED1 and OsNCED3 (Maruyama et al., 2014), suggesting that the ABA-dependent pathway plays roles in response to cold and drought in *Zea mays* ssp. *mexicana* L.

PP2C proteins are also reported to be involved in ABA-dependent regulations of stress induced responses, such as the 76 phosphatase 2C (PP2C-type) proteins which have been identified from *Arabidopsis*. Most of these (PP2C-type) proteins fall into 10 groups (A–J) (Schweighofer et al., 2004). ABA signaling can be mediated by type 2C protein phosphatases (PP2Cs) such as HAB1 and ABI2, which inhibit stress-activated SnRK2 kinases (Nakashima et al., 2009). When directly comparing the expression levels under cold with those under drought conditions in *Zea mays* ssp. *mexicana* L., four candidate PP2C genes, including c52482_g3, c52482_g6, c62830_g2, and c57868_g1, showed up-regulated patterns. A putative protein kinase SnRK1 (c57228_g1), however, was down-regulated only under cold stress. These results taken together suggest that PP2C protein is involved in ABA-dependent signaling and affects the regulation of cold induced responses in *Zea mays* ssp. *mexicana* L.

Previous studies on *Zea mays* ssp. *maize* L. transcript profile under cold, salt, and drought stresses indicate that the abundance of bioactive GAs was reduced and that the negative regulatory factor DELLAs were accumulated (Shan et al., 2013), suggesting a negative effects of gibberellin in stress induced responses. This is in disagreement with what we observed in cold-stressed *Zea mays* ssp. *mexicana* L. Genes for the GAs receptor GID1L2 precursor (c47232_g1), the GA 2-beta-dioxygenase (c57017_g1, c33506_g1 and c57017_g2) and the GA20 oxidase (c63822_g4, c49254_g1 and c56760_g2) that are involved in GAs synthesis or signaling were up-regulated by cold, suggesting a positive role of gibberellin in this subspecies in response to cold stress. Thus, GAs could differentially regulate the responses to cold in these two close *Zea mays* subspecies. Future comparative study will provide better understanding of the molecular basis of the GA associated response to cold in *Zea mays*.

In summary, this study provided an overview of the transcriptome of *Zea mays* ssp. *mexicana* L., and the changes of transcript abundance in response to cold or drought stress. Less than a half of the reported *Zea mays* proteome have homologs in *Zea mays* ssp. *mexicana* L., indicating that specific regulation networks or mechanisms exist in these close relatives. DEGs responsive to cold or drought conditions serve as candidates for further study on tolerance related gene networks in *Zea mays* ssp. *mexicana* L. The effects of drought and cold reported here have arisen from a limited range of potential types and severities of stress. Thus, it should be noted that a greater range of treatments for (e.g., timing, severity, frequency, source) need to be examined in future studies to provide more clues for understanding the adaptation and tolerance mechanisms in this species.

## Author contributions

XL conducted the bulk of experiments, data analysis, and wrote the paper draft. XZ and YC participated in the treatments of experimental materials. MZ and DM contributed to data interpretation and writing. CY and SL were responsible for experimental design, data interpretation and writing.

## Funding

This research was supported by the Guangdong Science and Technology Department (2015A020209155) and the National Natural Science Foundation of China (U1201212).

### Conflict of interest statement

The authors declare that the research was conducted in the absence of any commercial or financial relationships that could be construed as a potential conflict of interest.

## Supplementary material

Supplemental File 1 **Distribution of the length of transcripts and unigenes**.

Supplemental File 2 **GO classification of annotated unigenes**.

Supplemental File 3 **Blast against maize and *Arabidopsis* protein database**.

Supplemental File 4 **Differentially expressed genes in different samples**.

Supplemental File 5 **GO enrichment analysis by direct comparing cold with drought stresses**.

Supplemental File 6 **Primers used in qRT-PCR**.

Supplemental File 7 **GO enrichment analysis and classify of DEGs**.
