# Supplementary material for: RNA-seq Analysis of Cold and Drought Responsive Transcriptomes of Zea mays ssp. mexicana L
Source: Front Plant Sci. 2017 Feb 7;8:136. doi: 10.3389/fpls.2017.00136 (PMC5293773; doi:10.3389/fpls.2017.00136)
Supplement: Supplemental File 5 — GO enrichment analysis by direct comparing cold with drought stresses. [file DataSheet5.docx]

**Supplemental file 1. Distribution of the length of transcripts and unigenes.**

**Distribution of length**

| **Sample/replicate** | **Raw reads** | **Clean reads** | **Clean bases (%)** | **Q20** | **GC%** |
| --- | --- | --- | --- | --- | --- |
| Control 1-1 | 40,305,567 | 36,128,683 | 4.52G | 95.35 | 54.76 |
| Control 1-2 | 40,305,567 | 36,128,683 | 4.52G | 92.29 | 54.7 |
| Control 2-1 | 37,982,847 | 34,737,380 | 4.34G | 95.34 | 55 |
| Control 2-2 | 37,982,847 | 34,737,380 | 4.34G | 92.4 | 54.93 |
| Cold 1-1 | 35,495,938 | 32,200,406 | 4.03G | 95.42 | 54.76 |
| Cold 1-2 | 35,495,938 | 32,200,406 | 4.03G | 92.39 | 54.68 |
| Cold 2-1 | 41,113,853 | 35,916,262 | 4.49G | 95.91 | 54.37 |
| Cold 2-2 | 41,113,853 | 35,916,262 | 4.49G | 92.82 | 54.32 |
| Drought 1-1 | 38,734,285 | 35,137,981 | 4.39G | 95.44 | 54.48 |
| Drought 1-2 | 38,734,285 | 35,137,981 | 4.39G | 92.5 | 54.41 |
| Drought 2-1 | 36,736,709 | 32,995,519 | 4.12G | 95.65 | 54.18 |
| Drought 2-2 | 36,736,709 | 32,995,519 | 4.12G | 92.4 | 54.11 |

The sample of this study contained 2 replications for 2 samples of three treatments.


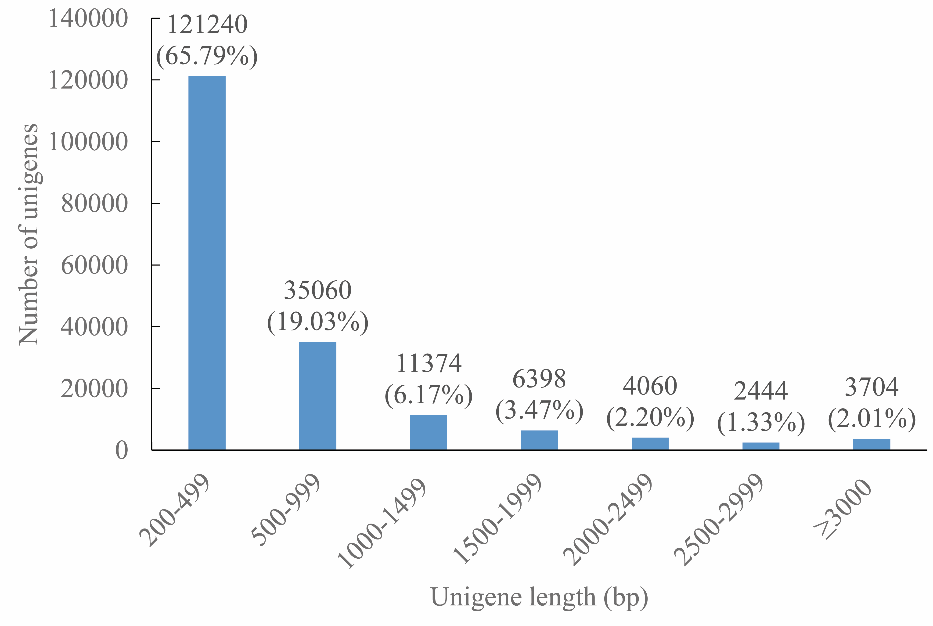


**Size distribution of the unigene sequences.**

The unigenes with different lengths (200 to 499 bp, 500 to 999 bp, 1000 to 1499 bp, 1500 to 1999 bp, 2000 to 2999 bp and ≥ 3000 bp) were shown, respectively. The percentages of the unigenes in each group out of the total unigenes (184,280) were also indicated.


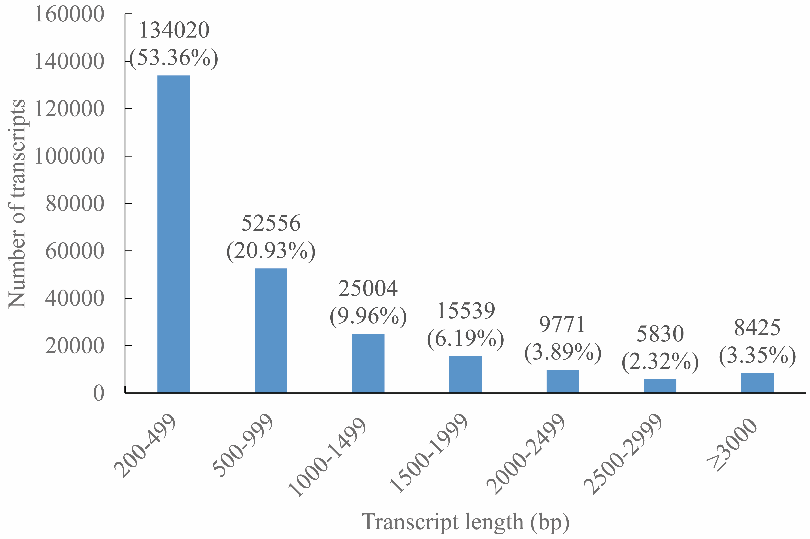


**Size distribution of the transcript sequences.**

The transcripts with different lengths (200 to 499 bp, 500 to 999 bp, 1000 to 1499 bp, 1500 to 1999 bp, 2000 to 2999 bp and ≥ 3000 bp) were shown, respectively. The percentages of the transcripts in each group out of the total transcripts (251,145) were also indicated.


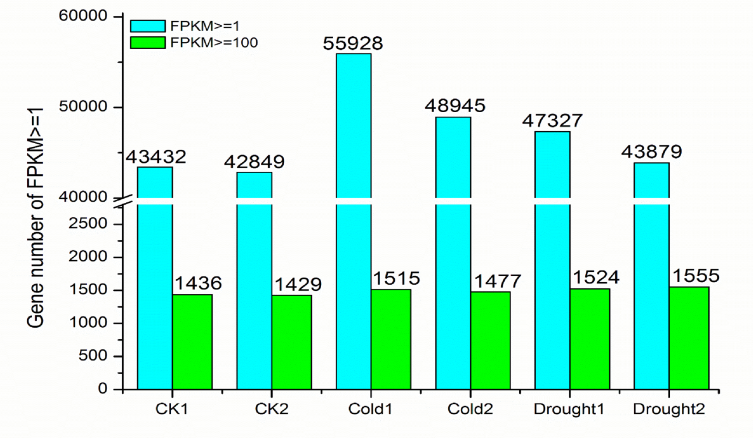


**The number of genes with differential expression found in the *Zea mays ssp. mexicana* L. expression libraries of duplicate samples (FPKM ≥ 1).**

Blue color means the number of genes of differential treatment with FPKM>=1; Green color means the number of genes differential treatment with FPKM>=100.
