# Supplementary material for: RNA-seq Analysis of Cold and Drought Responsive Transcriptomes of Zea mays ssp. mexicana L
Source: Front Plant Sci. 2017 Feb 7;8:136. doi: 10.3389/fpls.2017.00136 (PMC5293773; doi:10.3389/fpls.2017.00136)

**Supplemental file 5. GO enrichment analysis by direct comparing cold with drought stresses.**

| **GO term ID** | **Term_type** | **Term description** | **FDR** |
| --- | --- | --- | --- |
| GO:0009408 | P | response to heat | 4.00E-19 |
| GO:0009266 | P | response to temperature stimulus | 1.90E-12 |
| GO:0009628 | P | response to abiotic stimulus | 8.60E-11 |
| GO:0006950 | P | response to stress | 8.60E-11 |
| GO:0050896 | P | response to stimulus | 1.40E-06 |
| GO:0042542 | P | response to hydrogen peroxide | 3.90E-06 |
| GO:0000302 | P | response to reactive oxygen species | 5.70E-06 |
| GO:0009644 | P | response to high light intensity | 3.20E-05 |
| GO:0042221 | P | response to chemical stimulus | 4.30E-05 |
| GO:0016052 | P | carbohydrate catabolic process | 5.00E-05 |
| GO:0000272 | P | polysaccharide catabolic process | 7.30E-05 |
| GO:0009415 | P | response to water | 0.00015 |
| GO:0009414 | P | response to water deprivation | 0.00031 |
| GO:0004722 | F | protein serine/threonine phosphatase activity | 0.00052 |
| GO:0005983 | P | starch catabolic process | 0.00081 |
| GO:0016161 | F | beta-amylase activity | 0.002 |
| GO:0009251 | P | glucan catabolic process | 0.0029 |
| GO:0009738 | P | abscisic acid mediated signaling pathway | 0.0029 |
| GO:0006470 | P | protein amino acid dephosphorylation | 0.0029 |
| GO:0016160 | F | amylase activity | 0.0036 |
| GO:0006560 | P | proline metabolic process | 0.0037 |
| GO:0006355 | P | regulation of transcription, DNA-dependent | 0.0061 |
| GO:0009642 | P | response to light intensity | 0.0061 |
| GO:0009615 | P | response to virus | 0.0061 |
| GO:0045449 | P | regulation of transcription | 0.0061 |
| GO:0051252 | P | regulation of RNA metabolic process | 0.0065 |
| GO:0006350 | P | transcription | 0.0068 |
| GO:0006351 | P | transcription, DNA-dependent | 0.0068 |
| GO:0031326 | P | regulation of cellular biosynthetic process | 0.0068 |
| GO:0032774 | P | RNA biosynthetic process | 0.0072 |
| GO:0009889 | P | regulation of biosynthetic process | 0.0072 |
| GO:0006979 | P | response to oxidative stress | 0.0073 |
| GO:0071215 | P | cellular response to abscisic acid stimulus | 0.0076 |
| GO:0010556 | P | regulation of macromolecule biosynthetic process | 0.0082 |
| GO:0080090 | P | regulation of primary metabolic process | 0.0086 |
| GO:0010035 | P | response to inorganic substance | 0.0086 |
| GO:0009737 | P | response to abscisic acid stimulus | 0.0086 |
| GO:0019219 | P | regulation of nucleobase, nucleoside, nucleotide and nucleic acid metabolic process | 0.0086 |
| GO:0009416 | P | response to light stimulus | 0.0089 |

**Histogram of Gene Ontology (GO) enriched for differential expression unigenes.** (A) Ck vs Cold, (B) Ck vs. Drought and (C) Cold vs. Drought. The X-axis represents the GO subcategories, and the Y-axis (-log10 FDR value) shows he degree of significant enrichment.

A


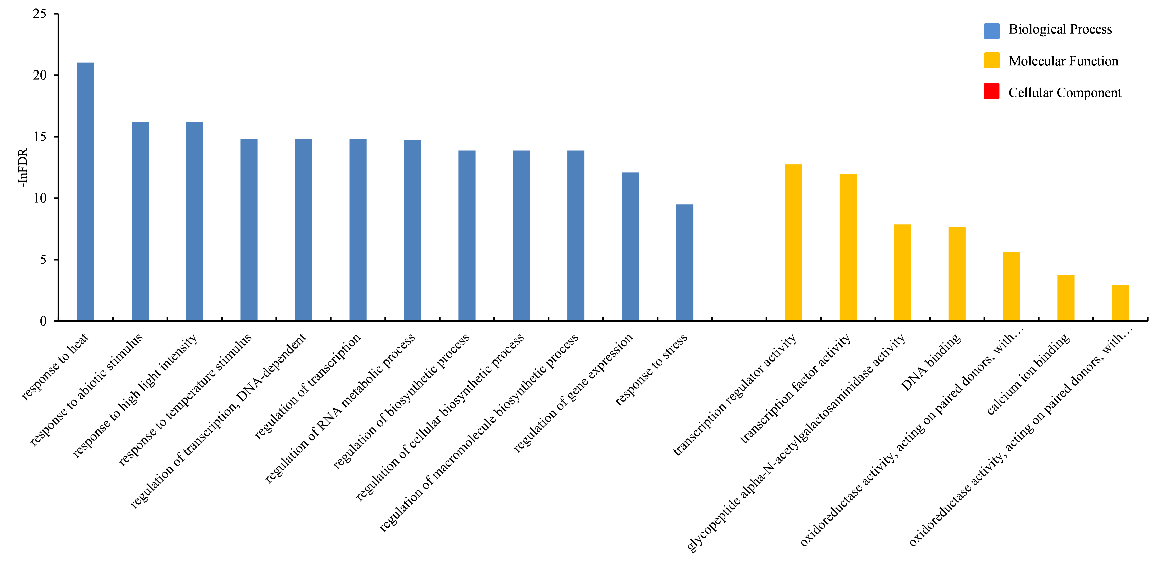


B


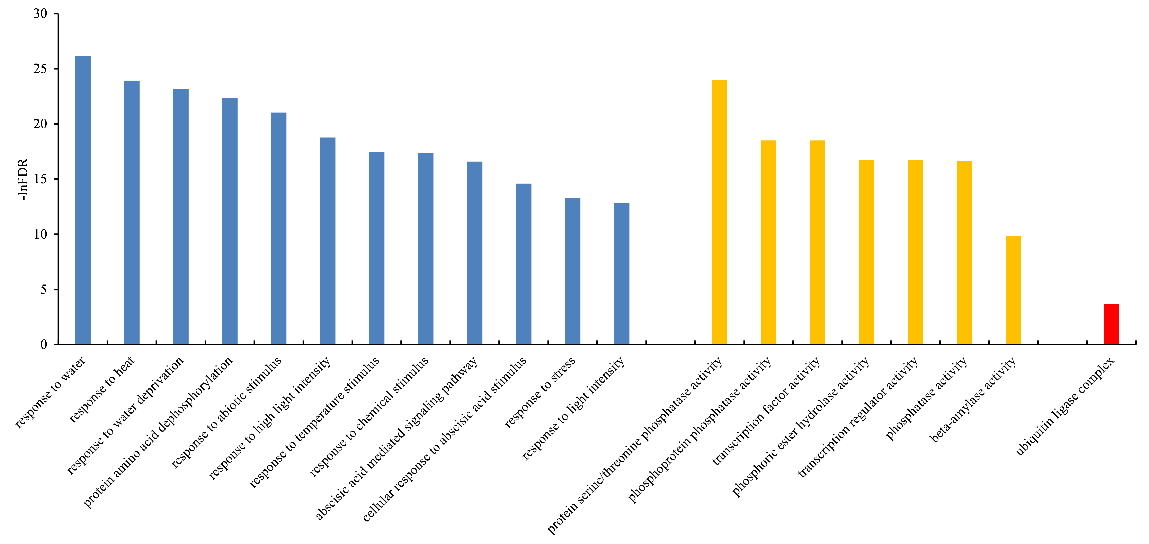


C


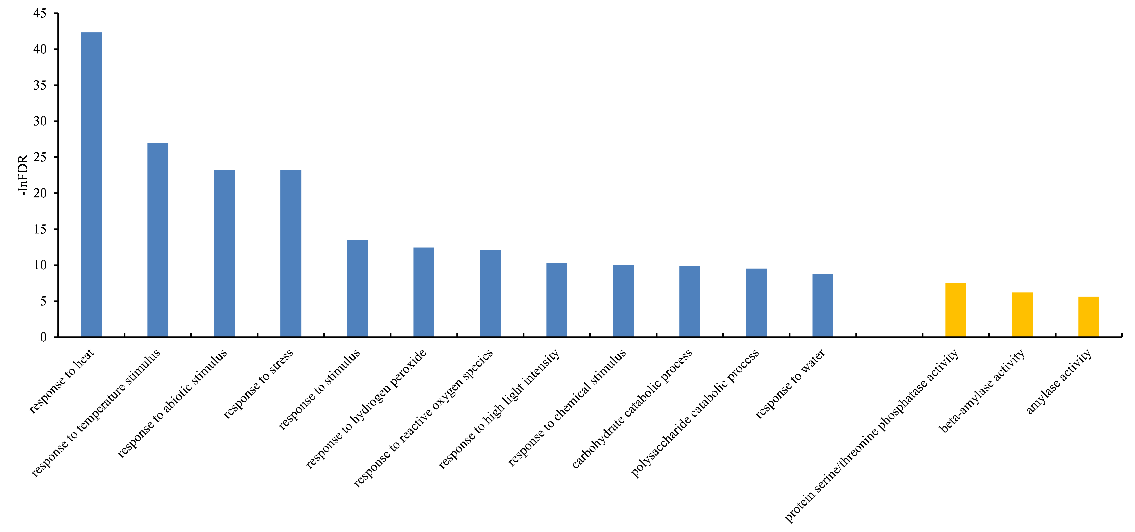

Supplement: Supplemental File 6 — Primers used in qRT-PCR. [file DataSheet6.docx]
