# Supplementary material for: RNA-seq Analysis of Cold and Drought Responsive Transcriptomes of Zea mays ssp. mexicana L
Source: Front Plant Sci. 2017 Feb 7;8:136. doi: 10.3389/fpls.2017.00136 (PMC5293773; doi:10.3389/fpls.2017.00136)
Supplement: Supplemental File 7 — GO enrichment analysis and classify of DEGs. [file DataSheet7.docx]

**Supplemental file 6. Primers used in qRT-PCR.**

| **Unigene ID** | **Primers** | **Description** |
| --- | --- | --- |
| c52482_g3 | F:TCACGAACGGCTTCAGGTAG | protein phosphatase 2C 3 [Arabidopsis thaliana]/uncharacterized [Zea mays] |
|  | R:TGCCTGCGTCGTTCTAATCA |  |
| c61291_g1 | F:TCCGTATCCATCCTTGTCCCT | PREDICTED: probable calcium-binding protein CML16 [Zea mays] |
|  | R:AGTATTTGCTGGGGTGGCAG |  |
| c59856_g7 | F:ACGGCGAAGAACCGGATAAG | heat shock factor protein HSF30 [Zea mays] |
|  | R:AAAAGAAGGCAAACCACACGG |  |
| c57152_g1 | F:TATTTCTTACCAATGTCGTCCCCAC | PREDICTED: transcription factor FER-LIKE IRON DEFICIENCY-INDUCED TRANSCRIPTION FACTOR-like [Zea mays] |
|  | R:TCACGTGCGCCATCTCTC |  |
| c51372_g1 | F:GGGCCAGCCTAACACGAA | PREDICTED: CBL-interacting serine/threonine-protein kinase 11 isoform X1 [Zea mays] |
|  | R:CAACAATAAACGGGTCGGCA |  |
| c54045_g1 | F:TGGAATGGAAGCGCAGACAC | PREDICTED: putative MYB DNA-binding domain superfamily protein isoform X1 [Zea mays] |
|  | R:TTCATCCGTGGCTTTGCCTA |  |
| c57309_g1 | F:AGAACAAGAATTGCCCACGC | PREDICTED: E3 ubiquitin-protein ligase EL5-like [Zea mays] |
|  | R:ACCTGCCAAGGATTTAGACGG |  |
| c54089_g2 | F:TCGTCCAGCTTTAGCCCTTC | heat shock protein 82 [Zea mays] |
|  | R:GGCTACGAGGTGCTGTTCAT |  |
| c56552_g1 | F:TCGTCTGGGGTTTCGTTTGA | PREDICTED: 17.0 kDa class II heat shock protein [Zea mays] |
|  | R:ACGTGGCATAACATGTCTCCTT |  |
| c58118_g1 | F:CGTATGGATAGGGCGTGAGG | PREDICTED: photosystem I reaction center subunit N, chloroplastic-like [Zea mays] |
|  | R:GCAGACAGCCTAAGCAAGCA |  |
| c58084_g2 | F:GGACAAGGAGCGACTCTAAGC | PREDICTED: probable protein phosphatase 2C 37 [Zea mays] |
|  | R:CAGAGACACAGGTAGCCACG |  |
| c60754_g2 | F:ACGTGTGGTGGATTCATGGT | homeobox-leucine zipper protein ATHB-6 [Zea mays] |
|  | R:TCTGTTTCCCGTCGATCCCT |  |
